# A Generalizable and Discriminative Learning Method for Deep EEG-Based Motor Imagery Classification

**DOI:** 10.3389/fnins.2021.760979

**Published:** 2021-10-22

**Authors:** Xiuyu Huang, Nan Zhou, Kup-Sze Choi

**Affiliations:** ^1^Centre for Smart Health, The Hong Kong Polytechnic University, Hong Kong, Hong Kong SAR, China; ^2^College of Control Engineering, Chengdu University of Information Technology, Chengdu, China

**Keywords:** electroencephalogram, motor imagery, convolutional neural networks, label smoothing, center loss

## Abstract

Convolutional neural networks (CNNs) have been widely applied to the motor imagery (MI) classification field, significantly improving the state-of-the-art (SoA) performance in terms of classification accuracy. Although innovative model structures are thoroughly explored, little attention was drawn toward the objective function. In most of the available CNNs in the MI area, the standard cross-entropy loss is usually performed as the objective function, which only ensures deep feature separability. Corresponding to the limitation of current objective functions, a new loss function with a combination of smoothed cross-entropy (with label smoothing) and center loss is proposed as the supervision signal for the model in the MI recognition task. Specifically, the smoothed cross-entropy is calculated by the entropy between the predicted labels and the one-hot hard labels regularized by a noise of uniform distribution. The center loss learns a deep feature center for each class and minimizes the distance between deep features and their corresponding centers. The proposed loss tries to optimize the model in two learning objectives, preventing overconfident predictions and increasing deep feature discriminative capacity (interclass separability and intraclass invariant), which guarantee the effectiveness of MI recognition models. We conduct extensive experiments on two well-known benchmarks (BCI competition IV-2a and IV-2b) to evaluate our method. The result indicates that the proposed approach achieves better performance than other SoA models on both datasets. The proposed learning scheme offers a more robust optimization for the CNN model in the MI classification task, simultaneously decreasing the risk of overfitting and increasing the discriminative power of deeply learned features.

## 1. Introduction

Brain–computer interface (BCI) has been raising interest from the research community. It provides an important way for the disabled to interact with the outside world without using any muscular movements (Wolpaw et al., 2002). This technology aims to recognize the user intentions based on the distinct patterns of neural events. Motor imagery (MI) is one of the crucial topics in the area of BCI, referring to a cognitive procedure of the motion imagination such as lifting left or right leg, without any actual moving actions (Ahn and Jun, 2015). The most popular technology to signalize such cognitive procedures is the electroencephalogram (EEG), being noninvasive and relatively easy to set up (Ahn and Jun, 2015; Ni et al., 2020; Zhang et al., 2020). The principle of the EEG-based MI-BCI system is to match the type of motion imagination and its corresponding EEG signals. Such matching systems have been practiced in a variety of applications, including speller (Rezeika et al., 2018), wheelchair (Kaufmann et al., 2014), and prosthesis (Vidaurre et al., 2016).

**Figure 1:**
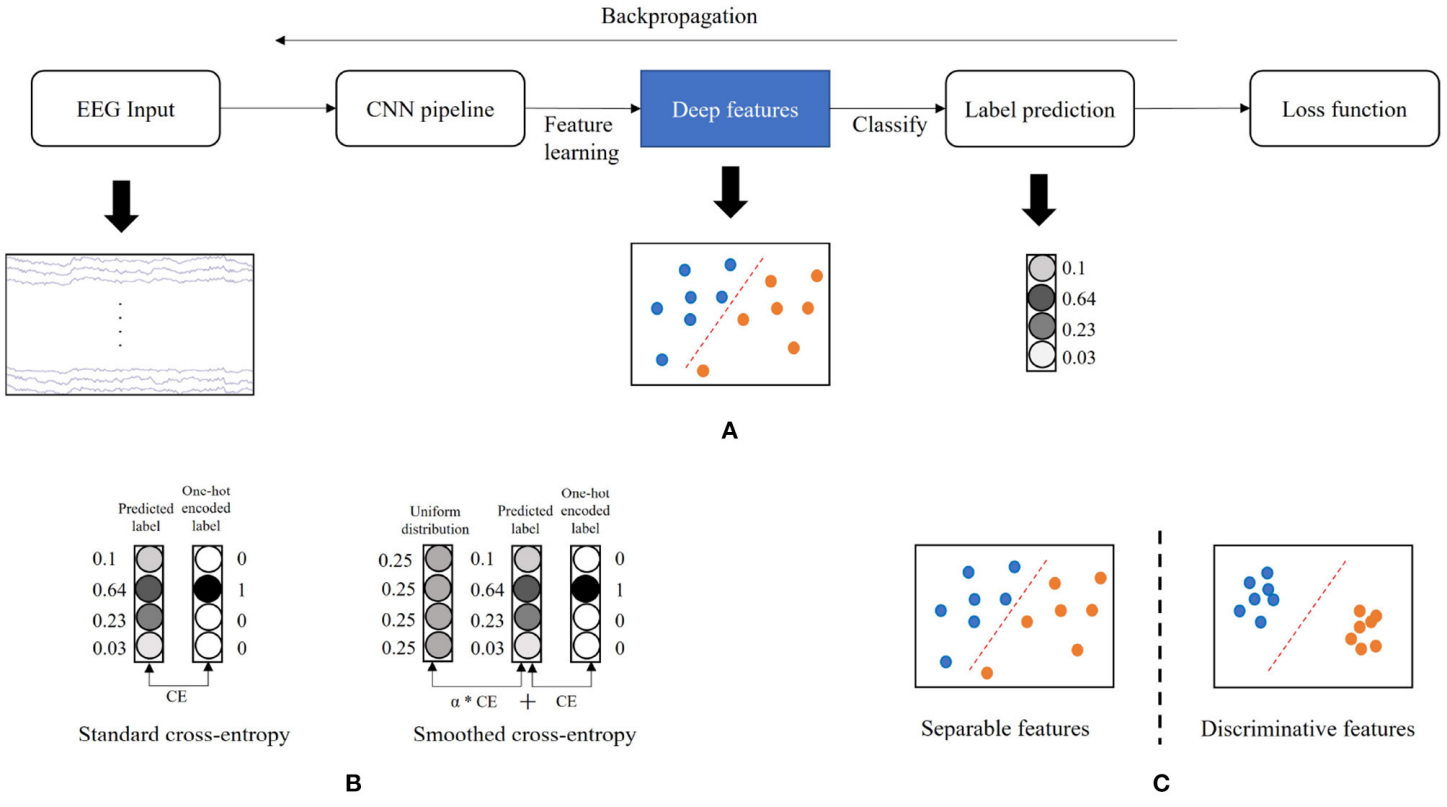
Illustration of the concepts in the study. **(A)** The common framework of convolutional neural network (CNN). **(B)** Label smoothing. **(C)** Deep feature distributions. The CE denotes the cross-entropy calculation. The α denotes the weight of the CE between predicted label and uniform distribution.

Accurate classification of EEG-MI pattern is one of the most decisive factors to the BCI performance but remains a significant challenge due to the low signal-to-noise ratio (SNR) characteristics of the EEG signal (Goldenholz et al., 2009; Zhang et al., 2019). Convolutional neural networks (CNNs) have been widely explored and achieved great success in the MI recognition area (Bashivan et al., 2015; Roy et al., 2019). It significantly pushes the boundary of the state-of-the-art (SoA) in classification accuracy compared to the conventional methods such as band power analysis (Martinez-Leon et al., 2015), independent component analysis (ICA) (Lee et al., 1999), and common spatial filter (CSP) (Ramoser et al., 2000). The most common framework of CNN is to perform feature generation and label prediction, learning deep features from raw EEG data by the CNN pipeline, then making label predictions based on the learned features (see Figure 1A).

The training of CNNs in the MI classification task is mainly guided by minimizing the cross-entropy (Hertz et al., 2018). This objective function is “greedy” and encourages the largest possible logit gaps, making the model less adaptive, and sometimes overconfident to its predictions (Szegedy et al., 2016). The model learns to assign a full probability to the ground-truth label for each training example, even though some noisy data are mixed in the training set (Müller et al., 2019). This overfitting phenomenon incredibly easily occurs when the training sample size is small. Coincidentally, the EEG data have a low SNR and contains much noise. In addition, the MI-BCI system is usually designated as subject dependent, so it usually has limited training data. To reduce the risk of overfitting (overconfidence) issue, we adopt a label smoothing technique introduced by Szegedy et al. (2016) in the training scheme of the MI classification. It computes a modified cross-entropy, called smoothed cross-entropy, not by using “hard” one-hot encoded labels such as [0, 0, 1, 0] from training data, but a weighted mixture of these hard labels with the uniform distribution (Figure 1B). Label smoothing alternatively encourages small logit gaps and prevent overconfident predictions. This technology has successfully increased the performance of CNN models across multiple tasks, including image classification (Szegedy et al., 2016), speech recognition (Chorowski and Jaitly, 2016), and machine translation (Vaswani et al., 2017). It is expected to benefit the model training in MI classification by tackling the overfitting problem, leading to a generalizable and adaptive CNN model.

In addition to ensuring the model's generalizability, we also aim to increase its discriminative power. As shown in the common framework of CNN (Figure 1A), the last fully connected layer acts as a linear classifier, and the cross-entropy only encourages the separability (Hertz et al., 2018) but does not guarantee the high discriminative characteristics, where features have both a large inter-class difference and a tight intra-class variation (Figure 1C). Therefore, the resulting features generated by the model trained via the cross-entropy are not sufficiently effective for the MI classification. To enhance the discriminative capacity of the deep features, we apply a center loss (Wen et al., 2016) for the model training. Specifically, the center of deep features is computed by their means in each class and updated across every epoch. The distances between deep features with their corresponding class centers are minimized at each training iteration. The parameters are optimized by jointly minimizing the cross-entropy and center loss. Intuitively, the cross-entropy forces the deep features from different classes to stay apart, and the center loss pulls the features belonging to the same class toward their centers. With joint supervision, we can concurrently enlarge the inter-class difference and reduce the intra-class variation so as to improve the discriminative power of deep features.

In this paper, we propose a novel training scheme for CNN-based model in the MI classification by using a combined loss with smoothed cross-entropy and center loss. The main contributions are as follows:

1. To our best knowledge, although structures of the CNN model are heavily investigated, this is the first attempt to use the proposed loss to help supervise training in the context of MI classification. With joint supervision of the smoothed cross-entropy and the center loss, both generalizable and discriminative model can be obtained for robust MI recognition.
2. We present extensive experiments on two famous MI public datasets, called BCI-competition IV-2a and IV-2b. Our new approach achieves superior performance compared to other SoA methods.
3. We also conduct an ablation study to demonstrate the effectiveness of the label smoothing and the center loss.

The remainder of the paper is organized as follows. In section 2, conventional and deep learning methods on MI classification are introduced. Section 3 describes the proposed approach. Sections 4 and 5 present the experiment result and analysis. Section 6 concludes the current study.

## 2. Related Works

A sophisticated feature extractor is the key to success in conventional methods for the MI classification task. One of the most frequently and widely used approaches is the common spatial pattern (CSP) (Pfurtscheller and Neuper, 2001; Yu et al., 2019). It tries to generate optimal spatial filters that have minimum or maximum variance between classes in a particular frequency band. The features used in the winner algorithm of the BCI competition IV are based on the filter bank CSP (FBCSP) (Ang et al., 2008) that finds a set of optimal spatial filters in multiple frequency bands. The Naive Bayes Parzen Window classifier using these features achieved an outstanding classification performance with an accuracy of 67.75% on the dataset IV-2a (Ang et al., 2008). After the competition, a novel method based on the support vector machine (SVM) with Riemannian covariance achieved a better performance (75.74%) for the same database (Hersche et al., 2018). In addition to these vector-based methods, matrix-form strategies such as the logistics regression classifier with a nuclear norm regularization (Zhou and Li, 2014), the rank-k SVM (Lal et al., 2004), and the support matrix machine (SMM) (Zheng et al., 2018) were also developed by multiple research groups. The leading edge of these methods is to directly process the 2-D MI EEG data on a matrix basis instead of stacking features as a vector input to a classifier, which preserves the informative structural patterns.

Deep learning (DL) models were also exploited to tackle the MI classification challenge. For instance, the multilayer perceptron (MLP) was proposed to generate nonlinear patterns from CSP features and also to substitute the SVM as a classifier for MI recognition (Kumar et al., 2016). Similarly, a channel-wise convolution with channel mixing (C2CM) was introduced to classify the spatial-temporal features generated by FBCSP (Sakhavi et al., 2018). Bashivan et al. (2015) converted the EEG waves into spectral topographies via short-time Fourier transform (STFT). These topographies were then fitted into CNNs for further transformation and classification. Tabar and Halici (2016) also used the STFT approach to extract spatial-temporal images as the feature input to the CNN-SAE model for classification. These feature input (FI) models still require complex feature generation from raw EEG data prior to the DL modeling. Several research groups investigate raw signal input (RSI) models to provide an end-to-end scheme for MI recognition to address this limitation. For example, the two most well-known RSI networks, EEGNet (Lawhern et al., 2018), and ConvNet (Schirrmeister et al., 2017), achieved competitive classification performance without using any pre-processing techniques. In general, although model architectures were heavily investigated, the neural network learning process did not receive too much attention from the MI community. Rather than figuring out a more sophisticated architecture, we propose a potentially efficient objective function for both generalizable and discriminative learning in the CNN-based model.

## 3. Method

This section first introduces the notations and definitions used in this work and describes the CNN architecture. Then, the novel proposed loss is presented in detail.

### 3.1. Definition and Notations

Assuming that the DL model input is on a per-trial basis, where the continuous EEG is segmented into labeled trials, we define the segmented trials of a subject as ${\left\{\left[x_{i},y_{i}\right]\right\}}_{i=1}^{n}$, where $x_{i}\in {\mathbb{R}}^{E\times T}$ represents *ith* of EEG trials recorded by *E* electrodes and *T* sampling time points. $y_{i}\in {\mathbb{R}}^{M}$ denotes the corresponding *i*^*th*^ labels of *M* classes. Let the ground truth distribution *p* over labels *p*(*y*|*x*_*i*_), and $\overset{M}{\underset{y=1}{\sum }}p\left(y|x_{i}\right)=1$. We also define a CNN-based model with θ that predicts label distribution *q*_θ_(*y*|*x*_*i*_), and certainly $\overset{M}{\underset{y=1}{\sum }}q_{\theta }\left(y|x_{i}\right)=1$. We are motivated to adopt the label smoothing technology and center loss to improve the generalizable and discriminative power of the CNN-based model.

### 3.2. Network Architecture

**Figure 2:**
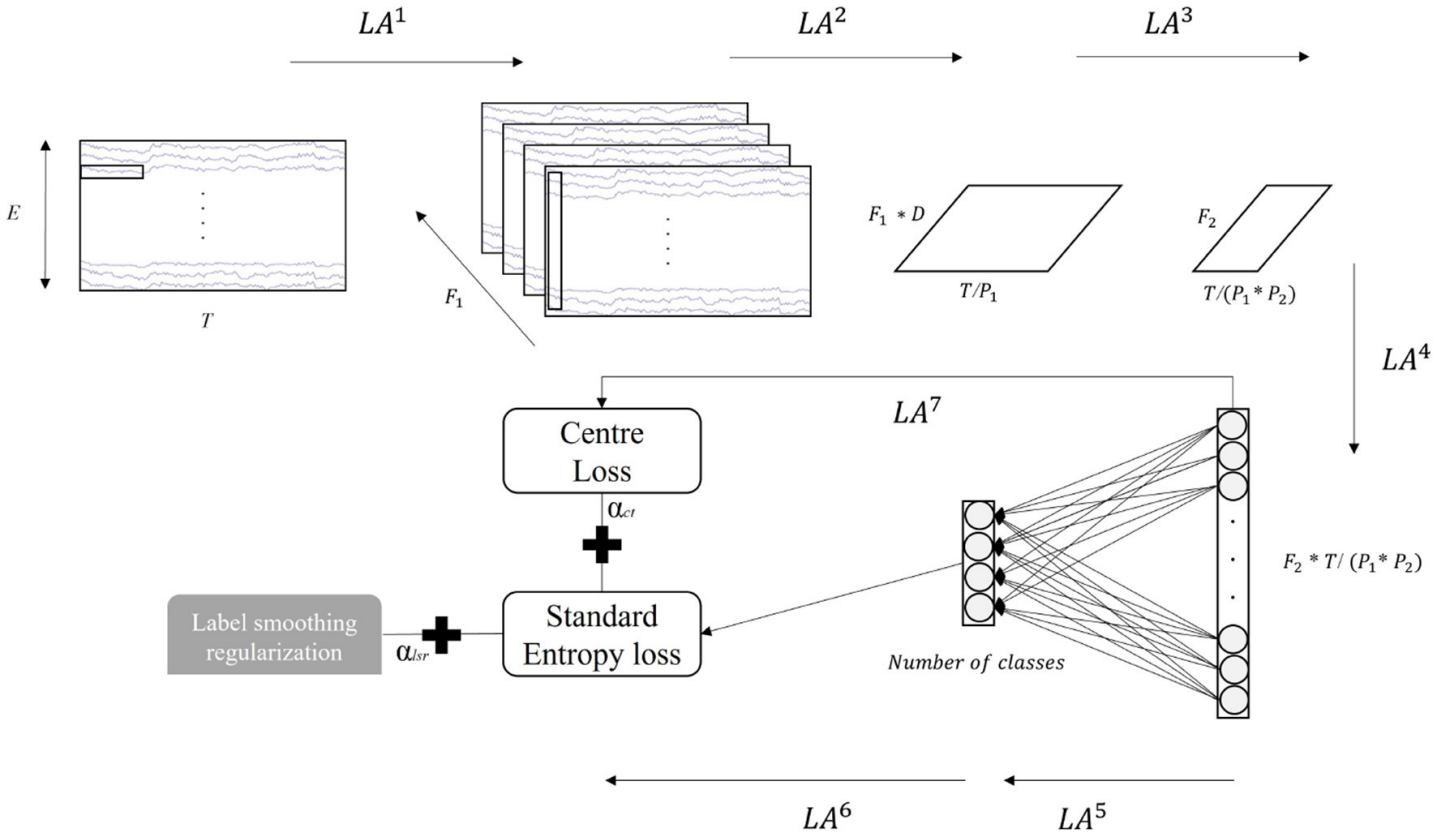
Illustration of the convolutional neural network (CNN)-based model. *T* = number of the timestamps, *E* = number of electricodes, *F*_1_ = number of filters in the temporal CNN, *D* = number of depthwise convolution output channels, *F*_2_ = number of spatial filters, *P*_1_ and *P*_2_ are sizes of the average pooling kernel, and α_*lsr*_ and α_*ct*_ are weights of label smoothing regularization and center loss.

In the current study, we inherit the CNN architecture of the EEGNet (Lawhern et al., 2018) but make two modifications, where the kernel size of the first temporal CNN filter (*LA*^1^) is decreased to attain temporal information above 8 Hz, as the alpha (8–12 Hz) and beta (12.5–30 Hz) band contain most relevant information of the motor imagery task (Wierzgała et al., 2018). The illustration of the network is displayed in Figure 2, and the details of each layer are presented in Table 1. The model begins with a 2D-CNN directly linked to the raw EEG data with a kernel size in (*K*_1_, 1) to capture temporal patterns in each electrode. A depthwise convolution layer with a kernel size of (1, *E*) is followed and utilized for spatial feature extraction. The separableConv2D with a kernel size in (*K*_2_,1) is then performed to gain deeper and more abstract temporal information across all electrodes. As shown in Table 1, it is noted that batch normalization (Ioffe and Szegedy, 2015), exponential linear unit (ELU) (Clevert et al., 2015) activation, and average pooling are sequentially followed after some of these convolutions for covariate shift avoidance (Bickel et al., 2009), nonlinear transformation, and dimension reduction, respectively. The deep feature generated by the CNN pipeline is then flattened as a vector (nodes) by a flatten layer. The vectors of each training batch are used to compute the center loss. The dense layer is subsequently connected to these nodes and acts as a classifier. The *softmax* function finally performs the estimation of the probability for each MI class. The cross-entropy between the probability estimation and the smoothed label represents the classification loss (standard cross-entropy + label smoothing regularization). The weighted sum of the classification and center losses supervises the training of the entire network.

**Table 1:** Architecture setting.

**Layer**	**Function**	**Filter**	**Kernel**	**Output shape**
*LA* ^1^	Input			*T, E*, 1
	Conv2d	*F*_1_	(*K*_1_, 1)	*T, C, F*_1_
	BatchNorm			
*LA* ^2^	DepthwiseConv2d	*F*_1_*D	(1, *E*)	*T*, 1, *F*_1_*D
	BatchNorm			
	ELU Activation			
	Average pooling		(*P*_1_, 1)	*T*/*P*_1_, 1, *F*_1_*D
*LA* ^3^	SparableConv2d	*F*_2_	(*K*_2_, 1)	*T*/*P*_1_, 1, *F*_2_
	BatchNorm			
	ELU Activation			
	Average pooling		(*P*_2_, 1)	*T*/(*P*_1_*P_2_), 1, *F*_2_
*LA* ^4^	Flatten			(T*F_2_)/(*P*_1_*P_2_)
*LA* ^5^	Fully connected			*number of classes*
*LA* ^6^	Softmax (CEL)			
*LA* ^7^	Lambda (CL)			1

*(1) T =number of the timestamps, E =number of electricodes, K_1_ = kernel size of the first CNN, D =number of depthwise convolution output channels, F_1_ = number of temporal filters, F_2_ = number of spatial filters, K_2_ = size of th kernel in the spatial filer, and P_1_ and P_2_ are sizes of average pooling kernels*.

*(2) CEL stands for cross-entropy computed by smoothed labels. CL stands for center loss. Lamda layer is a self-customize layer for the calculation of the center loss*.

### 3.3. Proposed Loss

To ensure both generability and discriminative power of the CNN-based model in the context of MI classification, we propose a combined loss that jointly optimizes the classification loss (e.g., cross-entropy computed by smoothed labels) and the center loss. Most models in previous studies performed the standard cross-entropy for the objective function defined as


(1) $$\begin{array}{l} L_{cl}=\sum_{i=1}^{n}H_{i}(p,q_{\theta })\\ \;=-\sum_{i=1}^{n}\sum_{y=1}^{M}p(y|x_{i})log\;q_{\theta }(y|x_{i}) \end{array}$$

Given the *p*(*y*|*x*_*i*_) is one-hot encoded in classification task where


(2) $$p(y|x_{i})=\left\{ \begin{array}{l} 1,\;y=y_{i};\\ 0,\;otherwise. \end{array}\right.$$

We can further reduce (1) as


(3) $$\begin{array}{l} L_{cl}=-\overset{n}{\underset{i=1}{\sum }}log\;q_{\theta }\left(y_{i}|x_{i}\right) \end{array}$$

For each training sample *i*, the *q*_θ_(*y*_*i*_|*x*_*i*_) is usually calculated by the *softmax* function as follows:


(4) $$q_{\theta }(y_{i}|x_{i})=\frac{exp(z_{y_{i}})}{\sum_{j=i}^{m}exp(z_{j})}$$

Here, *z*_*j*_ is the logit value or unnormalized log-probability for each label *j*. By using the one-hot ground-truth label, minimizing the objective function *L*_*cl*_ is equivalent to do the log-likelihood maximum. The maximum is not achievable with finite data, so it can only be estimated in the case when *z*_*y*__*i*_ >> *z*_*j*_ for all *j* ≠ *y*_*i*_ (e.g., the logit of the ground-truth label is much larger than all other logits) over the training dataset (Szegedy et al., 2016). In such a case, the model learns to classify every training sample correctly with a confidence of nearly 1, which is the signal of overfitting. This phenomenon relatively easily occurs in the scenario that the MI EEG task often only contains a small sample size of training data.

We adopt the label smoothing mechanism where a noise distribution *u*(*y*|*x*) is added to the one-hot ground truth label to prevent the model from having overconfidence and to reduce the risk of overfitting. Then, the new ground truth label distribution is $p^{\prime }\left(y|x_{i}\right)=\left(1-\varepsilon \right)p\left(y|x_{i}\right)+\varepsilon u\left(y|x_{i}\right)$, where ε is a weight factor, ε* ϵ* [0, 1]. By replacing *p*(*y*|*x*_*i*_) with $p^{\prime }\left(y|x_{i}\right)$ in (1) and (2), the new classification loss $L_{cl}^{\prime }$ computed by smoothed labels can be written as


(5) $$\begin{array}{l} L^{\prime }{}_{cl}=\sum_{i=1}^{n}\sum_{y=i}^{M}p^{\prime }(y|x_{i})\log q_{\theta }(y|x_{i})\\ \;=-\sum_{i=1}^{n}\sum_{y=i}^{M}\left[(1-\varepsilon )p(y|x_{i})+\varepsilon u(y|x_{i})]q_{\theta }(y|x_{i}\right)\\ \;=(1-\varepsilon )\sum_{i=1}^{n}H_{i}(p,q_{\theta })+\varepsilon \sum_{i=1}^{n}H_{i}(u,q_{\theta }) \end{array}$$

The first half of (5) is the weighted standard cross-entropy *L*_*cl*_. Let the second half is weighted loss of label-smoothing regularization (*L*_*lsr*_), which penalizes the deviation of predicted label distribution *p* from noise distribution *u* with a relative weight ε/(1−ε). We set the noise as the uniform distribution *u*(*y*|*x*) = 1/*M* (Szegedy et al., 2016). Then, the *H*_*i*_(*u, q*_θ_) measures the dissimilarity between predicted label distribution *p* to uniform. Therefore, *L*_*lsr*_ heavily penalizes overconfident predictions and prevents poor generalization during the training.

Let the weight of *L*_*cl*_ is fixed as 1, and the relative weight ε/(1−ε) of *L*_*lsr*_ is redefined as α_*lsr*_. Therefore, $L_{cl}^{\prime }$ can be elaborated as


(6) $$\begin{array}{l} L_{cl}^{\prime }=L_{cl}+\alpha_{lsr}L_{lsr} \end{array}$$

In addition to the generalizability, we also try to ensure the discriminative ability of the deep learned features extracted by the CNN pipeline. Intuitively, simultaneously maximizing the inter-class distance and minimizing the intra-class variation is the fundamental strategy to keep features of different classes divisible. The cross-entropy minimization only assures to enlarge the inter-class distance, so we further employ a center loss to achieve intra-class variation reduction. We follow the equation proposed by Wen et al. (2016), and the center loss is defined as


(7) $$\begin{array}{l} L_{ct}=\frac{1}{2}\overset{n}{\underset{i=1}{\sum }}||x_{i}-c_{y_{i}}|{|}_{2}^{2} \end{array}$$

where *c*_*y*_*i*__ denotes the *y*_*i*_ center of the feature extracted by the CNN pipeline. Minimizing the distance between each deeply learned feature and its class center naturally decreases the intra-class variation. Finally, the objective function *L* is to jointly optimize the classification loss $L_{cl}^{\prime }$ and *L*_*ct*_, defined as


(8) $$\begin{array}{l} L=L_{cl}^{\prime }+\alpha_{ct}L_{ct} \end{array}$$

where α_*ct*_ is the weight of center loss. Based on (6), *L* can be re-defined as,


(9) $$\begin{array}{l} L=L_{cl}+\alpha_{lsr}L_{lsr}+\alpha_{ct}L_{ct} \end{array}$$

## 4. Experiments

The BCI competition IV-2a and IV-2b datasets are used to evaluate the proposed approach. These two datasets are publicly available. The people involved in the datasets have obtained ethic approval. Users can download the data for free for research and publish relevant articles, so the ethical review and approval were waived for the current study.

### 4.1. Dataset Description

The BCI competition IV-2a (Tangermann et al., 2012) was recorded from 9 healthy individuals (A01-A09) by 22 EEG and 3 EOG channels in a sample rate of 250 Hz. The cue-based paradigm is used during the data collection. It consists of four MI classes, including the imagination motions of the left hand, right hand, tongue, and both feet. Two separate sessions were implemented for each subject. Each session comprises a total of 4*72 (a single MI class) = 288 trials. For fair comparisons with other approaches, the same data division scheme as that in the competition was used in our experiment. The first section is for model training and the second for model testing. Only the 4-s temporal segment (from the start of the cue until the end of the MI) in each trial is used in our model. Given the 250 Hz sample rate, our experiment's training and testing data are on a 1,000-sample series basis.

The BCI competition IV-2b (Tangermann et al., 2012) was also collected from nine healthy people (B01–B09) but only with 3 EEG channels (C3, Cz, and C4) attached to the frontal cortex. The dataset comprises two MI classes, including left-hand and right-hand movement imagination based on a cue-based BCI paradigm. Five independent sessions were recorded for each individual. We also keep using the same data division as that in the competition. The first three sessions are for training, and the remaining two are for evaluation. The 4-s temporal interval, from the starting point of the cue until the end of the MI, is used as a trial in our experiment. Given the recording frequency of 250 Hz, each training or testing trial is also on a 1,000-point basis.

### 4.2. Experimental Setup

Our approach is performed on a Tesla V100-SXM2 GPU running on Google online platform (Colab). The CNN network and the proposed loss function are implemented by Keras. The model is trained with Adam (Kingma and Ba, 2014) optimizer using a learning rate of 0.001, mini-batch size of 64, and 750 epochs. According to the result of the ablation study stated in section 5.2, the loss weights α_*lsr*_ and α_*ct*_ are set as 0.5 and 0.5, respectively. Other hyperparameters of the model architecture are shown in Table 2.

**Table 2:** Hyperparameter settings.

**Hyperparameter**	**CNN for IV-2a**	**CNN for IV-2b**
*T*	1,000	1,000
*E*	22	3
*F*_1_	8	8
*F*_2_	16	16
*K*_1_	32	32
*K*_2_	16	16
*D*	2	2
*P*_1_	8	8
*P*_2_	8	8

To evaluate the effectiveness of the proposed approach, we compare our strategy against existing SoA methods, including two conventional approaches [a vector-based method, e.g., the competition winner algorithm FBCSP (Ang et al., 2008), and a matrix-based method, SMM (Zheng et al., 2018)], two compact well-known DNN methods [EEGNet (Lawhern et al., 2018) and shallow ConNet (Schirrmeister et al., 2017)], and one more DNN methods (DRDA, Zhao et al., 2020) with complex architecture. The evaluation metric is the classification accuracy (*acc*).

## 5. Results

### 5.1. Comparison With State-Of-The-Art Methods

The comparisons between the proposed methods and other models on the BCI competition IV-2a and IV-2b datasets are shown in Tables 3, 4, respectively. The classification accuracy of each subject and the average accuracy are reported in a subject-dependent basis (e.g., training and testing data are from the same subject) as the same as the competition data division scheme. The model that has the best performance for each subject is highlighted in boldface. Tables 3, 4 clearly show that the proposed strategy has the best classification accuracy for nearly all subjects on both datasets with a maximum of 14.16% (subject A05) better than the second-best on IV-2a and of 11.43% (B02) better than second-best on IV-2b. On the average level, the classification average accuracies of our method have improvements of around 5.33 and 3.54% on IV-2a and IV-2b compared to other SoAs. We conduct paired *t*-tests between our approach and other SoA strategies to verify if the improvements are statistically significant. The *p* values obtained from the tests are indicated in Table 5. We can see that all *p* values are <0.05, which advises that the performance improvements of our method against others are statistically significant. In addition, it also can be seen that the corresponding standard deviations (SDs) of our method are 10.07 and 8.54%, which are both the smallest SD on the respective datasets. This result suggests that our method is a more robust classifier in a subject-independent manner than other models. All these results, as mentioned earlier, demonstrate that the CNN model trained using the proposed loss provides a more accurate and stable classification outcome for the MI recognition task.

**Table 3:** Classification accuracies (%) obtained with the dataset BCI competition IV-2a.

**Methods**	**Subject**									**Average (SD)**
	**A01**	**A02**	**A03**	**A04**	**A05**	**A06**	**A07**	**A08**	**A09**	
FBCSP	76.00	56.50	81.25	61.00	55.00	45.25	82.75	81.25	70.75	67.75 (12.94)
SMM	81.94	59.38	81.60	62.85	59.03	49.36	86.11	77.78	78.47	70.72 (12.35)
EEGNet	85.76	61.46	88.54	67.01	55.90	52.08	89.58	83.33	**86.81**	74.50 (14.36)
ConNet	76.39	55.21	89.24	74.65	56.94	54.17	**92.71**	77.08	76.39	72.53 (13.42)
DRDA	83.19	55.14	87.43	**75.28**	62.29	57.15	86.18	83.61	82.00	74.75 (12.22)
**Ours**	**89.32**	**66.78**	**94.14**	74.56	**76.45**	**62.33**	86.28	**85.61**	85.23	**80.08 (10.07)**

*Highest values are highlighted in boldface*.

**Table 4:** Classification accuracies (%) obtained with the dataset BCI competition IV-2b.

**Methods**	**Subject**									**Average (SD)**
	**B01**	**B02**	**B03**	**B04**	**B05**	**B06**	**B07**	**B08**	**B09**	
FBCSP	70.00	60.36	60.94	**97.50**	93.12	80.63	78.13	92.50	86.88	80.01 (13.06)
SMM	67.81	51.79	53.44	93.31	82.81	74.69	72.19	82.50	75.62	72.68 (12.77)
EEGNet	68.44	57.86	61.25	90.63	80.94	63.13	84.38	93.13	83.13	75.88 (12.57)
ConNet	76.56	50.00	51.56	96.88	93.13	85.31	83.75	91.56	85.62	79.37 (16.27)
DRDA	81.37	62.86	63.63	95.94	93.56	88.19	85.00	95.25	**90.00**	83.98 (11.94)
**Ours**	**83.33**	**74.29**	**72.65**	96.09	**95.97**	**88.84**	**92.24**	**96.09**	88.16	**87.52 (8.54)**

*Highest values are highlighted in boldface*.

**Table 5:** Paired *t-*test (*p*-values) between our method and others.

**Model**	**IV-2a**	**IV-2b**
FBCSP	0.0002	0.0058
SMM	0.0005	0.0001
EEGNet	0.0443	0.0013
ConNet	0.0168	0.0228
DRDA	0.0120	0.0479

### 5.2. Ablation Result Analysis

Ablation studies are carried out to study the contributions of the label smoothing technique and center loss to the CNN modeling. The hyperparameter α_*lsr*_ controls the degree of the smoothness on the label, and α_*ct*_ dominates the intra-class variations of the deep features. They are both significant. Therefore, two experiments on dataset IV-2a are explored to investigate the sensitiveness of these two hyperparameters.

#### 5.2.1. Label-Smoothing Regularization

**Figure 3:**
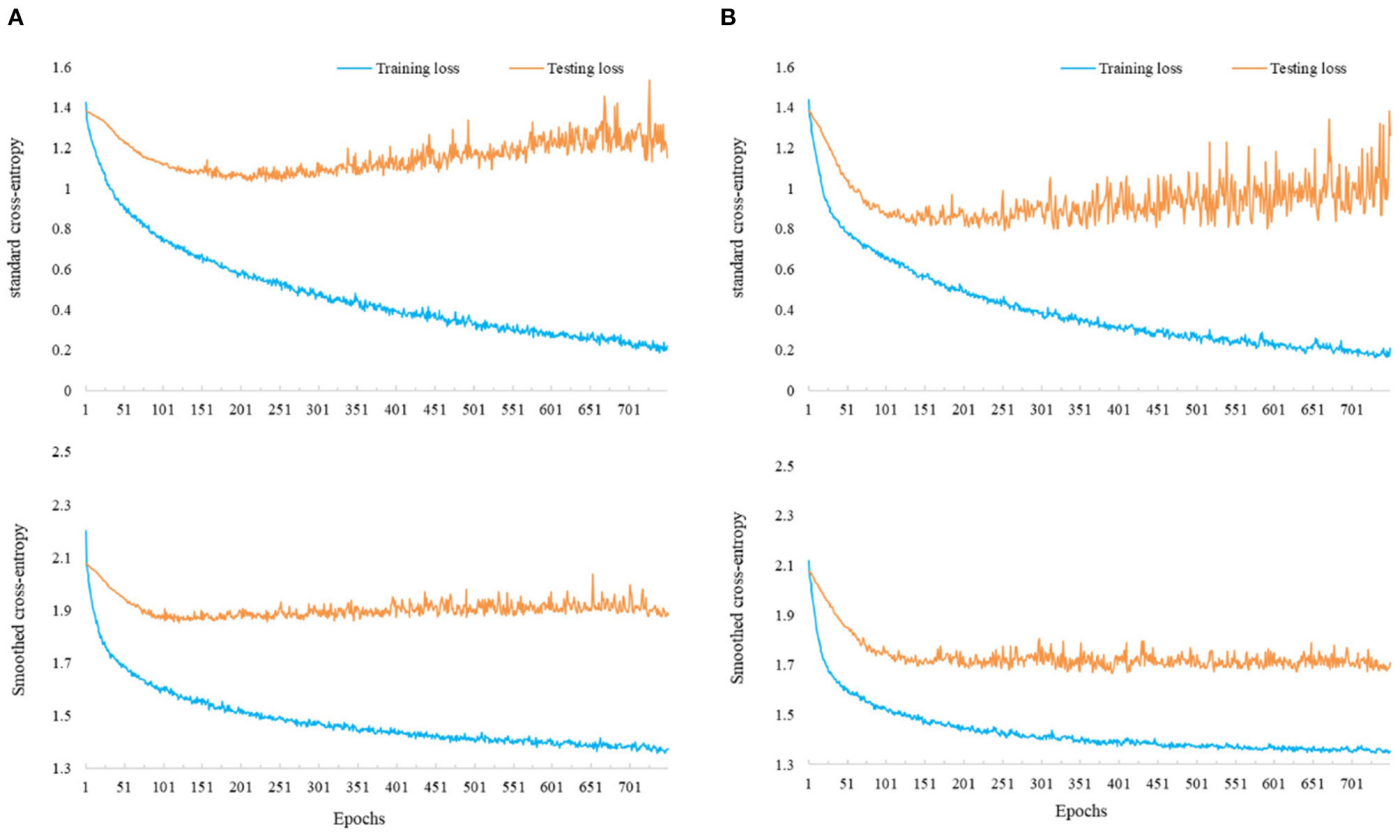
Training and testing losses of the models using standard cross-entropy (top) and smoothed cross-entropy (bottom). **(A)** Learning curve of the model on A02. **(B)** Learning curve of the model on A05.

In the first experiment, we fix the α_*ct*_ as 0, where no center loss is applied, and vary α_*lsr*_ from 0 to 1 (inclusive) to train different models. The verification accuracy for each subject and the averaged accuracy across these models are displayed in Table 6. From the average accuracy column, it is clear that only using the standard cross-entropy (in the case of α_*lsr*_ = 0) for the model training is not an excellent choice. Proper selection of α_*lsr*_ can improve the CNN-based model's verification accuracy on the MI recognition. Second, we also observe that the model performance remains relatively stable across different values of α_*lsr*_ in a range of [0.25, 1]. This phenomenon suggests that different levels of smoothness on labels may have a similar effect on the model performance in the MI area. Finally, it can be seen that the model has the most remarkable improvements on subjects A02 and A05, who have low predicting accuracy, by using the smoothed cross-entropy compared to using the standard one. We further visualize the training and testing loss during the optimization of these two subjects in Figure 3. It is recognized that the models using standard cross-entropy suffer from an overfitting issue where the training loss decreases at the beginning and flattens gradually, but the testing loss decreases at the beginning while increases after several epochs. On the contrary, the models using smoothed cross-entropy have a good learning curve. Both training and testing errors decrease at the beginning and then flatten until the end of optimization. Together with the verification accuracy improvement, this finding suggests that the label smoothing technique can degrade the influence of the overfitting on the CNN model in the MI classification.

**Table 6:** Classification accuracies (%) of models with different α_*lsr*_ values on the BCI competition IV-2a.

**α**_***lsr***_	**A01**	**A02**	**A03**	**A04**	**A05**	**A06**	**A07**	**A0**	**A09**	**Average**
0	84.34	52.65	**93.04**	66.67	52.90	57.00	87.73	81.55	80.3	72.91
0.25	80.78	**64.66**	92.67	67.54	69.57	**60.00**	85.92	80.07	78.79	75.58
0.5	**84.34**	61.48	90.84	**72.37**	**73.19**	59.53	85.56	**82.66**	**81.44**	**76.82**
0.75	82.92	63.60	90.11	66.23	72.46	53.49	85.92	82.29	81.44	75.39
1	81.14	62.19	91.94	64.04	69.20	58.14	**89.53**	80.07	82.58	75.43

*The α_ct_ is fixed as 0. The best accuracy for each subject is highlighted in boldface*.

#### 5.2.2. Center Loss

**Figure 4:**
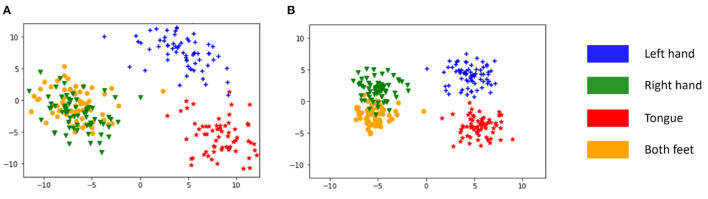
Visualization of deep feature distributions of subject A07 on dataset IV-2a under two conditions. **(A)** Model with α_*lsr*_ = 0.5 and α_*ct*_ = 0. **(B)** Model with α_*lsr*_ = 0.5 and α_*ct*_ = 0.5.

In the second experiment, we fix the α_*lsr*_ as 0.5 (performing the best in the first experiment) and vary α_*ct*_ in a range from 0 to 1 (inclusive) to train different models. The performances of these models are displayed in Table 7. When the α_*ct*_ is larger than zero, the center loss is activated, and the performance improves across almost all subjects and in the averaged level. This result suggests that the involvement of the center loss increases the discriminative power of the model. For a clear illustration and intuition, the principal component analysis (PCA) (Jolliffe and Cadima, 2016) is further employed to convert the high dimensional features of the second last layer in the model for subject A07 (randomly selected) into 2-D vectors. The distributions of these vectors are shown in Figure 4. It is clear that, without the center loss (Figure 4A), the deep features within each class are dispersive and have a larger intra-class variation. Alternatively, with the center loss in the joint supervision (Figure 4B), the features have both a compact intraclass distance and a clear interclass boundary. These results suggest that the center loss is beneficial to the discriminative feature learning for MI classification modeling.

**Table 7:** Classification accuracies (%) of models with different α_*ct*_ values on the BCI competition IV-2a.

**α**_***ct***_	**A01**	**A02**	**A03**	**A04**	**A05**	**A06**	**A07**	**A08**	**A09**	**Average**
0	84.34	61.48	90.84	72.37	73.19	59.53	85.56	82.66	81.44	76.82
0.25	87.54	64.31	94.14	73.25	74.64	**63.26**	86.64	80.07	81.82	78.41
0.5	**89.32**	**66.78**	**94.14**	**74.56**	**76.45**	62.33	86.28	**85.61**	**85.23**	**80.08**
0.75	89.32	66.43	93.77	73.68	71.38	60.47	89.17	84.5 0	85.23	79.33
1	87.9	59.72	93.77	71.49	74.64	57.67	**89.89**	83.39	81.82	77.81

*The α_lsr_ is fixed as 0.5. The best accuracy for each subject is highlighted in boldface*.

## 6. Conclusion

This paper proposes a new deep learning scheme for the CNN-based model in the MI classification. By jointly combining the smoothed cross-entropy with center loss, the robustness and discriminative power of the model can be highly enhanced for the classification. Extensive and systematic experiments are conducted to validate our strategy on two well-known benchmarks. Several suggestions have been made based on experimental findings. First, the label smoothing technique can degrade the overfitting issue caused by the scarcity and low SNR of the EEG data on the CNN model training. Second, the center loss along with the cross-entropy efficiently decreases the intra-class variance and thus increases the discriminative ability of the deep features by pulling them toward their corresponding latent class centers. It reduces the negative impact of the non-stationary characteristics of the EEG data on the MI classification task. Finally, the proposed loss offers a robust and discriminative training scheme for CNN-based modeling in the MI area. This phenomenon uncovers the fact that, in addition to sophisticated model structure development, implementing an efficient loss function for the learning guidance is also beneficial to model performance in MI recognition. This research has thus encouraged more attempts on the objective function innovation for the deep learning model in the MI field. It can be an interesting alternative for overcoming the bottleneck performance to the model architecture that has been heavily investigated.

## Data Availability Statement

Publicly available datasets were analyzed in this study. This data can be found at: http://www.bbci.de/competition/iv/.

## Ethics Statement

Ethical review and approval was not required for the study on human participants in accordance with the local legislation and institutional requirements. Written informed consent for participation was not required for this study in accordance with the national legislation and the institutional requirements.

## Author Contributions

XH conceptualized the study, performed the majority of the experiments and analyses, made the figures, and wrote the first draft of the manuscript. NZ and K-SC performed some experiments, updated the figures, performed the statistics, and edited the manuscript. All authors approved the submitted version.

## Funding

The work in this paper was supported in part by the Hong Kong Innovation and Technology Fund (MRP/015/18), the Hong Kong Research Grants Council (PolyU 152006/19E), and National Nature Science Foundation of China (Nos. 61802036 and 11901063).

## Conflict of Interest

The authors declare that the research was conducted in the absence of any commercial or financial relationships that could be construed as a potential conflict of interest.

## Publisher's Note

All claims expressed in this article are solely those of the authors and do not necessarily represent those of their affiliated organizations, or those of the publisher, the editors and the reviewers. Any product that may be evaluated in this article, or claim that may be made by its manufacturer, is not guaranteed or endorsed by the publisher.
